# Use of New Green Bitumen Modifier for Asphalt Mixtures Recycling

**DOI:** 10.3390/ma15176070

**Published:** 2022-09-01

**Authors:** Szymon Malinowski, Michał Wróbel, Lidia Bandura, Agnieszka Woszuk, Wojciech Franus

**Affiliations:** Department of Construction Materials Engineering and Geoengineering, Faculty of Civil Engineering and Architecture, Lublin University of Technology, Nadbystrzycka 40, 20-618 Lublin, Poland

**Keywords:** asphalt, biopolymer, asphalt mix, reclaimed asphalt pavement, sodium alginate

## Abstract

Nowadays, an increasing amount of reclaimed asphalt pavement (RAP) is being produced from the reconstruction and/or modernisation of asphalt pavements. It is necessary to recycle the obtained RAP according to principles of sustainable development. Therefore, this work includes the design of asphalt mixtures containing RAP with bio-derived modifier and evaluates their performance properties. Crosslinked sodium alginate was applied for bitumen modification. The studies were carried out for four different modifier contents, i.e., 1.0%, 2.5%, 4.0% and 5.5%, with and without crosslinking agent. On the basis of the binder test results, the optimal amount of the additive was found to be 2.5%. The nanostructure analysis for the base and modified binders indicated a dual crosslinked biopolymer effect. As a result of the bee structure size decrease, the binder softening effect was observed. The asphalt mix properties showed that application of biopolymer-modified binder is fully justified due to the functional parameters of the mixture, especially the increased resistance to water and frost by about 9%.

## 1. Introduction

Modern road engineering demonstrates the necessity and high validity of using waste materials from various industries in the production of asphalt mixes [1,2,3]. Moreover, the change of the road traffic structure causes intensified degradation of road surfaces that results in an increased generation of RAP (Reclaimed Asphalt Pavement), the application of which as a full-value material in the production of asphalt mixes meets the principles of sustainable development as the most convenient method of its use and management [4]. Due to its many environmental and economic benefits, RAP utilisation is currently being widely investigated by many researches and asphalt producers [5]. The challenge of RAP usage lies in achieving homogeneous mixture and satisfactory properties in combined binders including aged and virgin bitumen components. Despite many advantages of RAP utilisation, the mixed binder can negatively affect selected properties of asphalt mix, which is the main weakness of applying recycled material [6]. To improve the mechanical properties of asphalt mix containing RAP, it is necessary to use either softer binder, rejuvenators [7,8], or bitumen modified with synthetic polymers [9]. Since application of soft binder is impractical and economically unjustified in the case of higher RAP contents, extensive studies concerning the use of rejuvenators and conventional polymer-modified binders have been carried out by many researchers [10].

According to the literature, it is known that the long-term performance of RAP-containing asphalt mix can be upgraded by the addition of bitumen modified with synthetic copolymers, i.e., styrene-butadiene-styrene (SBS), styrene-butadiene-rubber (SBR) and poly-butadiene-rubber (PBR) [11]. Their application leads to decreased bitumen penetration, which may be considered as negative effect, further leading to over-stiffening of the asphalt mix. Daryaee et al. [9] evaluated the combination effect of waste polymer, soft binder and rejuvenator on recycled asphalt mix and demonstrated that the simultaneous application of different additives leads to softening of the aged binder and improves moisture damage resistance. Additionally, copolymer-modified bitumen is characterised by a higher softening point, which has a positive effect on rutting resistance.

In recent years, a new trend related to the usage of renewable compounds of biological origin, i.e., biomass or biopolymers, for asphalt modification has been observed [12,13]. The biopolymers are synthesised by microorganisms, plants, trees or other biological organisms [14], and they do not cause harmful effects on the environment or humans [15]. Pèrez et al. [16] applied waste containing vegetal lignin biopolymer for partial bitumen substitution in asphalt mix. The studies showed an improvement of permanent deformation and water resistance of asphalt concrete containing 20% lignin. The study described by Yuanita et al. [17] indicated that lignin biopolymer addition enhanced interactions between polymer and bitumen as a result of the chemical reaction of OH groups present in biopolymer with asphalt components by ether (-C-O-C-) linkage formation. Therefore, lignin can be successfully applied as a coupling agent in polypropylene-modified bitumen. The main chemical compound derived from living organisms widely applied in road technology is cellulose. do Vale et al. [18] reported that both pure cellulose fibres as well as lignocellulose fibres derived from coconut shell have a positive impact on moisture resistance of asphalt mix. Moreover, the acetylated cellulose addition reduces bitumen temperature sensitivity, while simultaneously maintaining its elastic behaviour [19]. The cellulose addition also influences basic bitumen properties by reducing its penetration, as well as raising its softening point and dynamic viscosity [20].

The biopolymers group also includes alginates, which consist of randomly linear unbranched chains of α-1-guluronate (G block) and β-d-mannuronate (M block) residues in the form of hydrophilic, anionic and colloidal heteropolysaccharides [21]. Depending on alginate source, their structure involves G and M blocks arranged as repeating (GGGG or MMMM blocks), alternating (GMGM), or random units [22]. The molecular weight of commercial alginates ranges from 32,000 to 400,000 g/mol [23]. To date, alginates have found many applications in the road industry, mainly in the form of capsules. Multi-cavity Ca-alginate capsules filled by water/sunflower oil emulsions have been applied in preparation of self-healing asphalt mixtures. During the pavement life cycle, the capsules release oil into the asphalt matrix, causing softening and having a rejuvenating effect on the binder [24]. The results presented by Tabaković et al. [25] indicated that even application of pure calcium alginate fibres caused an increase in asphalt mix stiffness modulus. The investigation of asphalt mix crack at the microscale showed the healing effect of the capsules, even without the rejuvenator. The self-healing effect in porous asphalt concrete [26] and asphalt mastic [27] was also observed. To date, however, bitumen has been modified with alginates only in the form of capsules filled with rejuvenator to obtain a microcrack self-healing effect. This paper involves a new approach to the application of alginates for direct bitumen modification in order to restore RAP binder properties The presented solution also has a positive environmental aspect resulting from the use of a non-toxic, biodegradable bio-based modifier. In contrast to the solutions reported in the literature, in this work, we demonstrate the possibility of crosslinking the biopolymer in a liquefied asphalt binder environment, leading to softening. Such a solution is very important in an era of special protection of the environment and natural resources, as well as the increasing amount of RAP production.

Taking into consideration the concern for non-renewable resources, it is extremely important to develop road pavement technologies with the application of RAP and innovative organic materials. One of the possible pathways of progress in this matter is the production of asphalt mix containing RAP and biopolymer-modified bitumen. Therefore, the primary goal of this study is to evaluate the effect of sodium alginate as biopolymer and its mixture with epichlorohydrin as crosslinking agent on bitumen properties, as well as on the properties of asphalt mix containing RAP. The research presented in this work makes it possible to analyse the possibility of using biopolymer-modified asphalt and biopolymer/crosslinking agent-modified asphalt and to estimate the softening effect of these modifiers on aged asphalt in RAP.

**Figure 1:**
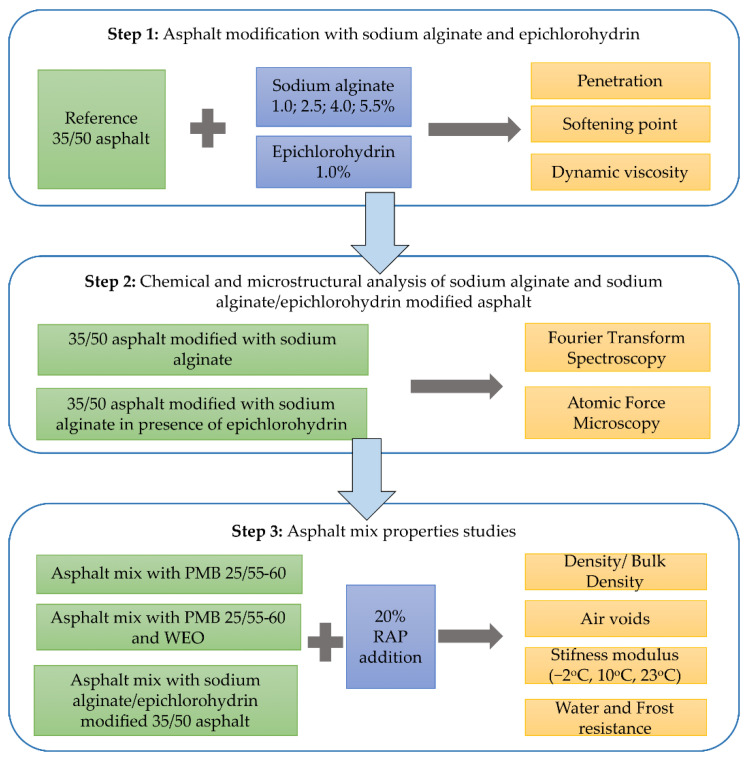
Research plan.

A graphical scheme of the research plan is shown in Figure 1.

## 2. Materials and Methods

### 2.1. Materials

Paving-grade bitumen 35/50 was selected as a base material for modification. The second bitumen that was applied in the third step of the study was polymer-modified bitumen type 25/55-60, further abbreviated as PMB 25/55-60. The properties of the applied bitumens are shown in Table 1. Aggregates used in the study were dolomites acquired from Piskrzyn mine, whereas the filler was ground limestone from Bukowa mine. The waste engine oil applied to one of the mixtures in Step 3 of the study was of the 5W40 type. The sodium alginate purified from algae with empirical formula (C_6_H_7_NaO_6_)n was purchased from Biomus and used without further purification. Extra-pure-grade (>99%) epichlorohydrin (1-chloro-2,3-epoxypropane) with empirical formula C_3_H_5_ClO, molecular weight 92.52 g/mol and dynamic viscosity 1.03 mPa·s was obtained from Fluka Analytical.

### 2.2. Sodium Alginate- and Sodium Alginate/Epichlorohydrin Mixture-Modified Bitumen Preparation

Based on the 35/50 bitumen, the biopolymer-modified bitumens containing 1%, 2.5%, 4%, 5.5% *w/w* sodium alginate in relation to the weight of the binder were prepared. To study the effect of epichlorohydrin, samples containing 1% epichlorohydrin in relation to the weight of the binder were prepared. The 35/50 bitumen modification procedure involved its heating at a temperature of 160 °C, and then the addition of an appropriate amount of sodium alginate followed by homogenisation by mechanical stirring while maintaining the temperature in the range of 180 ± 5 **°**C. The sodium alginate/epichlorohydrin-modified bitumen samples were prepared by the addition of appropriate amount of sodium alginate and epichlorohydrin followed by homogenisation by mechanical stirring, maintaining the temperature in range of 180 ± 5 **°**C.

### 2.3. Asphalt Mix Design

In this study, three different mixtures were designed for asphalt concrete layers with a maximum aggregate size of 16 mm, which is in line with the current Polish technical requirements; WT-2 2014. The addition of RAP was 20% in all mixtures. In the reference asphalt mix (PMB), a commercially available asphalt modified with a synthetic polymer called PMB 25/55-60 was used. The properties of this asphalt are summarised in Table 1. In the asphalt mix designated WEO-PMB, the commercial asphalt PMB 25/55-60 was also used. However, in order to soften the aged asphalt present in the RAP, the WEO additive was used, which was dosed directly into RAP in an amount of 7% of the bitumen mass contained in RAP. In the BPMB mix, a 35/50 asphalt modified with a biopolymer was used, the percentage content of which had been previously optimised, and the results obtained are presented in Section 3.1. The mixtures were designed using the grading envelope method. The gradation of the aggregates was determined using the dry sieving method, whereas in the case of the filler, the air jet sieving method was applied. The composition of both mineral and asphalt mixtures is presented in Table 2. The target bitumen content in the designed mixtures was 4.5%; however, considering the share of the RAP binder, the final addition of the bitumen was equal to 3.3%.

### 2.4. Test Methods

The penetration of bitumen was tested at 25 °C using a semi-automatic penetrometer in accordance with the EN 1426:2009 standard. The softening point test of bitumen was determined in water using the ring and ball method according to the EN 1427:2009 standard, whereas dynamic viscosity tests were carried out in a Brookfield viscometer at 160 °C according to the ASTM D 4402 standard.

In the case of asphalt mixture testing following test procedures and standards were applied: density—EN 12697-5:2018; bulk density (SSD)—EN 12697-6:2018; air void content—EN 12697:8-2018; stiffness modules in Nottingham Asphalt Tester—EN 12697-26:2018. For the testing of stiffness, the following Poisson’s ratios and temperatures were adopted: 0.4 for 23 °C; 0.3 for 10°C; 0.25 for −2 °C. Measurements were carried out on three Marshall samples for every mix, each in two positions (90° angle of rotation). The determination of the indirect tensile strength ratio (ITSR), which corresponds to water and frost resistance, was performed according to the EN 12697-12:2018 standard with one freeze–thaw cycle according to AASHTO T283 [28].

For FT-IR spectra analysis, solutions at a concentration of 0.33 g/mL were prepared using analytical-grade tetrachloroethylene (C2Cl4) with a purity of >99.9%. Then, 200 µL of the as-prepared solution was dropped onto 300 mg of dried KBr and left at a temperature of 80 °C for 20 min to evaporate the solvent. The FT-IR spectra of the analysed samples were collected using a NICOLET 380 spectrometer applying the DRIFT technique from 4000 to 600 cm^−1^ using 1024 scans and at a resolution of 4 cm^−1^.

AFM (NanoScope V AFM (Veeco, New York, NY, USA)) was used to depict changes in bitumen binder topography (scan resolution was 256 × 256 pixels and 384 × 384 pixels). Nanoscope Analysis ver. 1.40 software (Veeco, New York, NY, USA) was used for data processing.

## 3. Results and Discussion

### 3.1. Determination of Optimal Biopolymer Dosage

**Figure 2:**
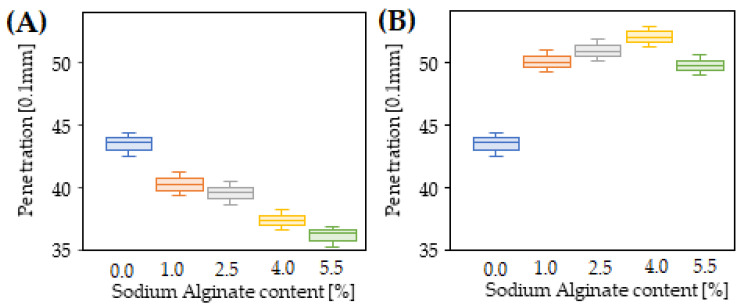
Penetration test results of 35/50 bitumen modified with sodium alginate (**A**) and 35/50 bitumen modified with sodium alginate in the presence of a crosslinking agent (**B**).

**Figure 3:**
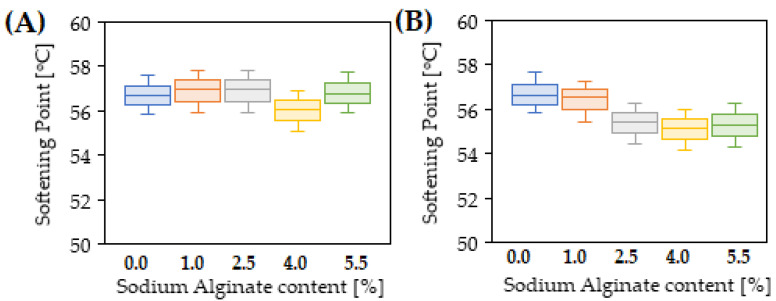
Softening point test results of 35/50 bitumen modified with sodium alginate (**A**) and 35/50 bitumen modified with sodium alginate in the presence of a crosslinking agent (**B**).

**Figure 4:**
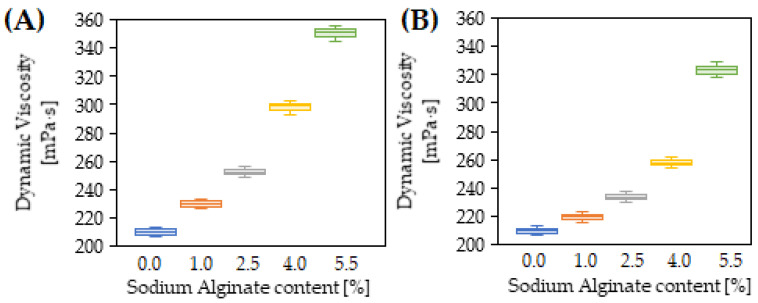
Dynamic viscosity test results of 35/50 bitumen modified with sodium alginate (**A**) and 35/50 bitumen modified with sodium alginate in presence of crosslinking agent (**B**).

**Figure 5:**
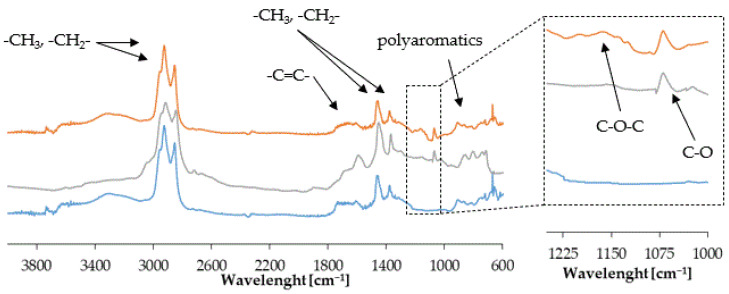
Infrared spectra of the control bitumen (blue line), bitumen modified with biopolymer (grey line), and bitumen modified with biopolymer in presence of crosslinking agent (orange line).

The results of penetration tests, softening point and dynamic viscosity of 35/50 biopolymer-modified bitumen are presented in Figure 2, Figure 3, Figure 4 and Figure 5.

The penetration of the reference 35/50 bitumen was 43.5 (0.1 mm). As a result of bitumen modification with sodium alginate (Figure 2), a decrease in penetration was observed in the range from 3.1 to 7.3 (0.1 mm). Simultaneously, the greater the amount of biopolymer that was used, the larger the change was. A similar trend was also observed for bitumen modified with cellulose, which also belongs to the group of biopolymers [20]. It is worth noting that the bitumen modified with synthetic SBS polymer used in this study (PMB 25/50-60) is also characterised by quite low penetration, equal to 38 (0.1 mm) (Table 1). Therefore, it can be concluded that modification with the biopolymer has similar effects on bitumen hardening to modification with the synthetic SBS polymer and, despite the decrease in penetration, no increase in stiffness of the produced bitumen mix should be expected.

The use of a crosslinking agent in the form of 1% epichlorohydrin caused an increase in penetration in comparison to samples of biopolymer-modified bitumen, as well as control 35/50 bitumen. The obtained softening effect ranged from 9.8 (0.1 mm) for bitumen modified with 1% biopolymer additive to 13.6 (0.1 mm) for bitumen modified with 4% sodium alginate. The slight decrease in penetration observed for bitumen containing 5.5% sodium alginate and 1% crosslinking agent may be due to an excess of biopolymer, consequently leading to the presence of non-crosslinked molecules, the presence of which causes an increase in penetration, as discussed in the paragraph above. Considering that the biopolymer-modified bitumen is dedicated to asphalt mixes containing RAP, the determined penetration values indicate the possibility that an additional softening effect can be achieved on hard RAP binder. Similar results have also been observed in previous works in which imidazoline [29] and WEO [30] were used as rejuvenators. Therefore, it is expected that the softening effect will also be maintained in RAP binder, resulting in a decrease in stiffness and an increase in the low temperature resistance of the prepared asphalt mix.

The softening point values of 35/50 road bitumen and PMB 25/50-60 were 56.4 °C and 68 °C, respectively. Figure 3 shows that the addition of the modifier in the form of sodium alginate resulted in slight changes in the softening point of 35/50 bitumen within the range of measurement error of +0.6 °C to −0.4 °C. These results indicate that the modification of the 35/50 bitumen with sodium alginate did not have a negative effect on the softening point, which directly affects the possible service temperature conditions and resistance to permanent deformation of the pavement. The use of a crosslinking agent in the modification process slightly changed the softening point by an amount from +0.2 °C to −1.2 °C compared to the results obtained for the 35/50 bitumen control sample. Regarding the results obtained for the samples of biopolymer-modified bitumen, a decrease in the softening point after using epichlorohydrin ranging from 0.4 °C to 1.4 °C was observed. This indicates that the application of the crosslinking agent also does not have a significant effect on the softening point of the bitumen binder, although the softening effect of bitumen was achieved. Due to the lower probability of permanent deformations in the road surface, these results should be considered to be favourable. The softening of the bitumen without simultaneously affecting the softening point indicates that biopolymer usage for bitumen modification leads not only to physical, as with standard rejuvenators, but also chemical and nanostructural changes, which are analysed in Section 3.2. The lack of change in the softening point after both bitumen modification with biopolymer and its mixture with a crosslinking agent should be considered to be beneficial in view of the reports on the binder properties after the addition of typical rejuvenators. Across the literature, the use of rejuvenators in the form of WEO [30] or imidazoline [29] has resulted in an increase in bitumen penetration with a simultaneous softening point decrease for both 35/50 and polymer-modified bitumen.

The viscosity of the reference 35/50 bitumen was 210 mPa∙s. After modification with sodium alginate, it increased, ranging from 230 mPa∙s to 350.5 mPa∙s, depending on the amount of sodium alginate used. The greater the amount of biopolymer that was used to modify the binder, the greater the observed increase in viscosity. As is seen in Figure 5, the addition of 1% sodium alginate caused an increase in the viscosity of the binder by 20 mPa∙s. Then, along with increasing the amount of the biopolymer by 1.5% each time (from 2.5% to 5.5%), the following increases in viscosity were obtained: 30.5 mPa∙s, 37.5 mPa∙s and 52.5 mPa∙s, respectively. The increase in bitumen viscosity following the addition of biopolymer in the form of cellulose was also observed by Eskandarsefat et al. [20]. In comparison, the dynamic viscosity of PMB 25/50-60 bitumen was 594 mPa∙s, which is then reflected in the required operating temperatures. The use of a synthetic polymer in the bitumen modification increases its elasticity, especially at low temperatures. However, it increases the viscosity of the binder, which helps to reduce the rate of permanent deformation growth at high service temperatures. On the other hand, an increase in binder viscosity requires the use of higher operating temperatures to provide an appropriate coating of the aggregate with bitumen, thus guaranteeing the durability of the resulting connection between them. The necessity of using higher operating temperatures adversely affects the environment as it necessitates the emission of more pollutants. As indicated in Figure 4, bitumen modified with sodium alginate has a significantly lower viscosity than bitumen modified with SBS polymer, which generally positively affects the production process of bituminous masses.

Bitumen modified with the biopolymer in the presence of the crosslinking agent had a higher dynamic viscosity than the reference 35/50 bitumen samples. Figure 4 shows that the viscosity increased with increasing percentage of sodium alginate. For biopolymer contents of 1 to 4%, a linear increase in viscosity from 9.5 mPa∙s to 23.0 mPa∙s can be observed. When this value is exceeded, and 5.5% sodium alginate is introduced into the binder, a sharp increase to 65.5 mPa∙s can be observed. It is noteworthy that, compared to the results obtained on bitumen samples modified with the biopolymer alone, the use of a crosslinking agent caused a decrease in viscosity ranging from 10.5 mPa∙s for 1% sodium alginate to 40 mPa∙s when using a sodium alginate content of 4%. The trend towards a decrease in the viscosity of the modified bitumen after the use of a crosslinking agent will have a positive impact on the process of producing bitumen mixes, where a sufficiently low viscosity of the binder must be achieved to provide appropriate coating of the aggregate by the bitumen. It is estimated that the difference in production temperature of asphalt mixtures when using SBS polymer-modified binder and biopolymer-modified binder will be 10–15 °C, which would contribute to a reduction in the atmospheric emissions of hazardous compounds, fumes and aerosols [31].

### 3.2. Biopolymer-Modified Bitumennanostructure Analysis

#### 3.2.1. Fourier-Transform Infrared Spectroscopy

The FTIR spectra of r the reference and modified 35/50 bitumen samples are pictured in Figure 5. All of the investigated spectra contain peaks characteristic of bituminous binder components. The two sharp bands at wavelengths of around 2900 cm^−1^ and 2840 cm^−1^ are characteristic of vibrations of the -CH*_3_* and -CH*_2_*- groups of saturated aliphatic hydrocarbons, respectively [32]. In addition, the band at around 1450 cm^−1^ was also attributed to the deformation vibrations of the -CH*_3_* group [33]. At the wavelength region of about 1600 cm^−1^, a peak is visible originating from the stretching vibrations of the C=C bonds [34] of the benzene rings commonly occurring in bitumen components. Aromatic -C-H vibrations are also visible in the wavelength region between 900 cm^−1^ and 700 cm^−1^, as multiple bands [35]. The FTIR spectra of sodium alginate-modified bitumen involves the addition of a peak at 1062 cm^−1^ corresponding to elongation of C-O bonds [36] occurring in the structure of sodium alginate. This confirms the presence of modifier in the bitumen. In the FTIR spectra of bitumen modified with sodium alginate and crosslinking agent, a wide band at a wavelength of about 1150 cm^−1^ is present, which was attributed to C-O-C linkage [37]. This group of atoms is characteristic of the epoxy structures present in the epichlorohydrin molecule.

#### 3.2.2. Atomic Force Microscopy

**Figure 6:**
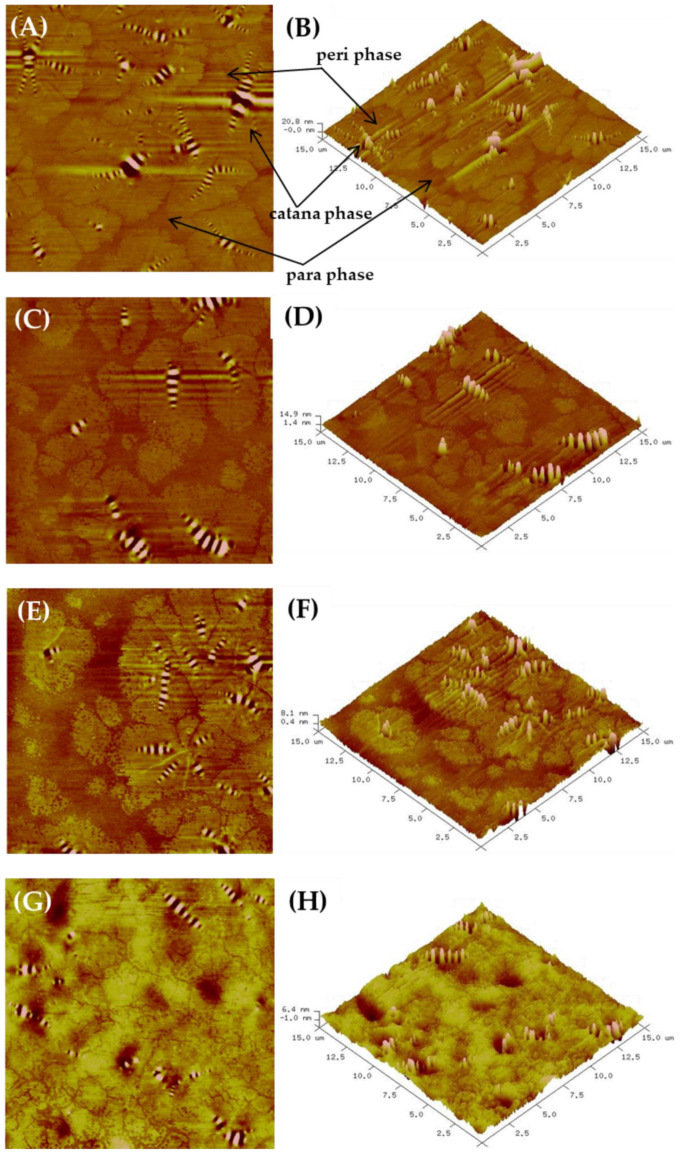
AFM topographical images of the control bitumen (**A**,**B**), bitumen modified with biopolymer (**C**,**D**), bitumen modified with biopolymer in presence of crosslinking agent (**E**,**F**), and PMB (**G**,**H**).

According to the theory of colloid bitumen structure [38], asphaltenes with high molecular weight are surrounded by polar components that form micelles dispersed in oil fraction, called maltenes [39]. In recent years, the AFM technique has become popular for the investigation of bitumen microstructure. The typical topography of binders consists of three different phases: (1) the catana phase, also known as bee structures that are suspected to be asphaltenes, (2) the peri phase, which surrounds the catana phase, and (3) the para phase, which is a smooth matrix of maltene fraction [40]. Figure 6A–F show the presence of bee structures in the matrix of both of the analysed samples. The topographical analysis carried out for a reference area of 15 × 15 µm revealed that the microstructure of the 35/50 bitumen involves bee structures with different sizes in the range of 1.31–3.64 µm. They often form dimeric or more branched clusters, consisting of elements of similar or different sizes. Additionally, smaller, single, and randomly distributed bee structures can be observed with diameters ranging from 0.61 to 0.97 µm. The addition of 2.5% biopolymer caused noticeable changes in the binder topography. Figure 6C,D show that biopolymer-modified bitumen exhibits a lower number of bee structures, with smoother shape, and with diameters ranging from 1.04 to 3.03 µm, in comparison to the non-modified binder. They form single agglomerates rather than branched clusters, and their protrusions are smaller in height and depth than in the case of non-modified binder. Supposing that they are derived from the asphaltene fraction, this indicates that the presence of sodium alginate without crosslinking agent can promote their agglomeration. This leads to bitumen hardening, as was also found in the penetration studies reported in Section 3.1. As can be seen in Figure 6E,F, the addition of crosslinking agent during bitumen modification is responsible for the formation of more bee structures with smaller diameters, ranging from 0.99 to 3.02 µm. They are finer and more heterogeneously distributed than in the non-modified binder or the binder modified with alginate. The observed bee structures form condensed agglomerates with clearly defined peri and para phases. Additionally, the formed protrusions are significantly smaller in height and depth, but the peri phase seems to exhibit a similar nature to that of the condensed bee structures (recognised by its height and colour). Decreases in bee structure size indicate a softening effect, which is assumed to be connected with the physical dissolution [41,42] of high-molecular-weight asphaltenes with crosslinked biopolymer molecules. The heating of 35/50 bitumen, as well as the addition of RAP binder, leads to an increase in the distance between bitumen components as a result of the weakening of π-π intermolecular interactions occurring between the benzene rings of the plane asphaltene molecules. Therefore, the sodium alginate crosslinked with epichlorohydrin can be successfully incorporated between planar asphaltene structures, thus limiting their re-aggregation during binder cooling. In consequence, it can be supposed that the formation of smaller asphaltene aggregates (dimers, trimers or tetramers) is responsible for the increase in bitumen penetration and the restoration of its primary properties.

### 3.3. Asphalt Mixtures Properties

#### 3.3.1. Volumetric Parameters

**Figure 7:**
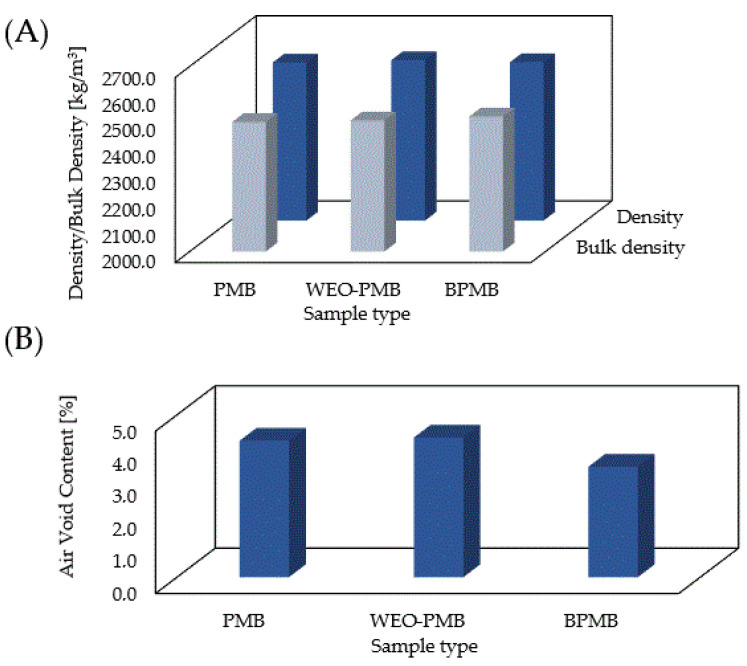
Density/bulk density (**A**) and air void content (**B**) of the PMB, WEO-PMB and BPMB mixtures.

The results of the volumetric properties are presented in Figure 7. The average value of the maximum density of the reference asphalt mixture was 2598 kg/m^3^, while the bulk density was 2490 kg/m^3^. The application of bitumen modified with a biopolymer and crosslinking agent had no effect on the density of the asphalt mixture. This was an expected outcome, as the amounts of modifiers used were small, and had no considerable effect on the density of the manufactured binder, which was comparable to that of bitumen PMB 25/50-60. However, the bulk density increased and, consequently, the air void content decreased. This was a result of the improved workability and compactibility of the asphalt mixture with biopolymer-modified bitumen. Similar results were obtained using bio-binder [43]. Taking into account the fact that the reference mix was compacted at a temperature that was 10 °C higher, due to the type of bitumen used, the achieved improvement in compactibility should be considered to be significant. As the void content increased with decreasing compaction temperature, it can be concluded that a content of 4.2% (as in the reference mix) could be achieved by lowering the compaction temperature of the samples with the biopolymer-modified bitumen. Such a procedure has been shown to provide additional environmental and economic benefits [44]. Conversely, insufficiently compacted asphalt layers would be more susceptible to the effects of water and frost, as well as intense bitumen oxidation, increased stiffness, and accelerated loss of service life [45].

The void content of the asphalt mix with synthetic bitumen and WEO addition was comparable to the value obtained for the reference mixture. However, the bulk density results showed an improvement in compactibility. This was due to the softening of the bitumen contained in the RAP and the fact that the decrease in viscosity of aged and new bitumen after the addition of WEO [30] is associated with a decrease in the amount of large molecules and carbonyl functional groups [46].

#### 3.3.2. Stiffness Modulus

In asphalt mixtures with RAP and rejuvenators, stiffness modulus testing is important [47]. It allows a preliminary assessment of the asphalt mixture resistance to both low and high temperatures. Asphalt mixture becomes stiffer under the influence of binder oxidation processes, and is more susceptible to low temperature, leading to premature degradation [48]. Hence, lower stiffness at negative temperatures is expected [45]. On the other hand, asphalt mixtures with a high stiffness modulus at positive temperatures tend to have better resistance to permanent deformation [49].

The average values of stiffness modulus are presented in Table 3. The reference PMB mixture had the highest value of stiffness modulus at both negative and positive temperatures. The following values were obtained: 6249 MPa at 23 °C, 11,518 MPa at 10 °C, and 19,743 MPa at −2 °C. As expected, the addition of WEO resulted in a decrease in the stiffness of the mixture, regardless of the test temperature. These results confirm the validity of using waste oils as softening additives for reclaimed asphalt pavement mixtures [50]. Asphalt mixtures with biopolymer-modified bitumen also exhibited a lower stiffness than the reference mix, which was a result of the effect of the biopolymer on the properties of the bitumen contained in the RAP. The test results conducted at 23 °C suggest a decrease in resistance to permanent deformation, which is characteristic of various types of rejuvenators [51]. On the other hand, an analysis of the results at a negative test temperature reveals an improvement in low temperature resistance obtained with the addition of WEO, as well as when using the bitumen modified with biopolymer. The sodium alginate used in combination with the crosslinking agent not only modified the base bitumen, in a similar way to that observed for the synthetic polymer, but also acted as a regenerating additive, improving the properties of the oxidised bitumen contained in the RAP. The formation of C=O and S=O groups as a consequence of bitumen ageing processes led to its hardening. Therefore, a decrease in their content is one way to restore the primary properties of the bitumen. As has been reported in the literature, a softening effect can also be observed as a result of chemical reactions between oxidised bitumen components and rejuvenating agent. Kuang et al. [52] reported that oxygen atoms possessing two pairs of unshared electrons are able to react with the oxygen-contained functional groups of binder components. As can be seen in Figure 1, the sodium alginate contains many -OH groups, and therefore it is highly probable that it can successfully restore the original properties of the RAP binder as a result of nucleophilic addition reaction.

#### 3.3.3. Water and Frost Resistance

**Figure 8:**
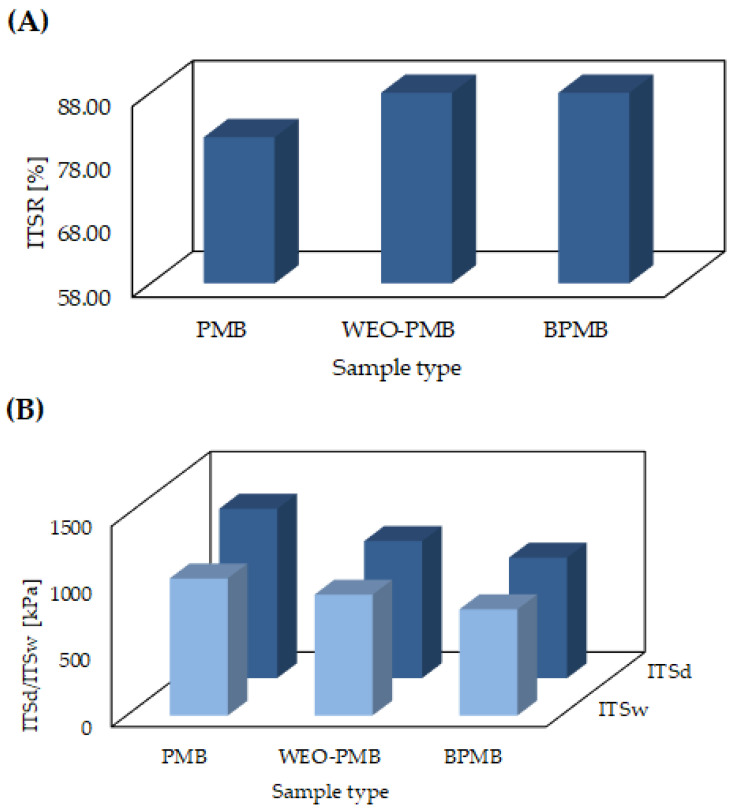
ITSR (**A**), ITSw and ITSd (**B**) of the PMB, WEO-PMB and BPMB mixtures.

The results of the strength properties together with water and frost resistance testing are presented in Figure 8. The reference asphalt mix had the highest average tensile strength values of 1268 kPa for dry and 1026 kPa for wet specimens, respectively. After application of the WEO rejuvenator, the strength decreased by 19.1% and 11.8%, respectively. In studies carried out by Mamun et al. [53] and Zaumanis et al. [7], the same trend was observed. The lowest strength was observed for samples prepared with the addition of biopolymer. Similar results were obtained for asphalt mixtures with latex-modified bitumen in a study by Suresh and Pal [54]. The difference in strength parameters may also be attributed to the fact that different base bitumens and different conditions were used to modify PMB bitumen with a synthetic polymer and in the case of biopolymer-modified bitumen preparation.

The asphalt mix with synthetically modified bitumen showed the highest decrease in water and frost resistance, with an ITSR value of 81%, whereas for the other two mixtures, the water and frost resistance was 88%. The observed increase in ITSR value confirms the high effectiveness of using the biopolymer as a modifier for bitumen. The obtained results also confirm proper mixing of the biopolymer-modified bitumen with the bitumen contained in RAP, as well as the achievement of proper bitumen–aggregate adhesion. Among other studies, Pasandín et al. [55] analysed binder–aggregate adhesion and obtained satisfying results for biopolymer addition in the form of waste cork. Peng et al. [56] investigated adhesion with the addition of lignin-based polyurethane, and it was found that the application of this biopolymer also increased the adhesion properties of the binder–aggregate system measured by FTIR, pull-off experiments and contact angle measurements.

## 4. Conclusions

This study was performed with an aim of recycling RAP with the application of a new polymer additive for bitumen. The biopolymer in the form of sodium alginate was tested with respect to its rejuvenating properties and its potential in the production of asphalt mix containing RAP. The bitumen tests, including penetration, softening point and dynamic viscosity, were carried out for different additive contents, ranging from 0% to 5.5%, with and without presence of epichlorohydrin as a crosslinking agent. Directly after testing the effect of the amount of modifier used in the bitumen, three different asphalt mixes were prepared, and their properties were tested.

The following conclusions were drawn:

- The sodium alginate modification of bitumen without crosslinking agent caused binder hardening through the formation of higher bee structures formed as a result of the formation of asphaltene aggregations.
- With the addition of 1% epichlorohydrin as a crosslinking agent, the binder softening effect was achieved. It is hypothesised that crosslinking of the biopolymer molecules takes place in the binder volume, resulting in the formation of structures with higher molecular weight. These structures can be incorporated between plane asphaltenes, limiting their aggregation and as a consequence reducing binder hardening. Secondly, the softening effect may also occur due to chemical reactions of sodium alginate -OH groups with oxygen atoms present in the structures of aged bitumen components.
- The observed decrease in viscosity of the binder modified with crosslinking agent provides appropriate aggregate coating during the asphalt mixture production process.
- The asphalt mix containing RAP with biopolymer-modified binder was characterised by better workability and compactibility, which had a direct impact on its volumetric properties, especially air void content.
- The stiffness modulus of the asphalt mix containing RAP and biopolymer-modified binder showed similar behaviour to the mixture with PMB rejuvenated by WEO at high temperature, whereas at low temperature, its behaviour was similar to the asphalt mix with PMB binder.
- A key finding of presented research was the enhancement of water and frost resistance (defined by ITSR indicator) in the asphalt mix with the biopolymer-modified bitumen.

The higher penetration value of the BPMB asphalt and the fact that the asphalt was softer translated into lower stiffness values of the asphalt mixtures obtained, while, at the same time, these mixtures were more susceptible to compaction, as is evident from the reduction in void content.

It is known from the literature that the use of WEO results in a softening of the asphalt, which translated into a reduced stiffness of the asphalt mixture at all measured temperatures.

The promising results of this study can contribute to the increased implementation of recycling in road construction. Further investigations will focus on evaluating the ageing process of RAP-rich asphalt mixes. This research is perfectly in line with the concept of the circular economy.

## Figures and Tables

**Table 1:** The properties of the used bitumen.

	Penetration [0.1 mm]	Softening Point [°C]	Dynamic Viscosity [mPa·s] (T = 160 °C)
35/50	43.5	56.4	210.0
PMB 25/55-60	38.0	68.0	594.0

**Table 2:** Mineral mix and asphalt mix composition.

Material	Percentage (% of Mass)	
	Mineral Mix	Asphalt Mix
Mineral filler	1.0	1.0
0/2 Dolomite	17.0	16.2
2/8 Dolomite	25.0	23.9
8/11 Dolomite	17.0	16.2
8/16 Dolomite	20.0	19.1
RAP	20.0	19.1
Bitumen	-	3.3

**Table 3:** Average stiffness moduli of the PMB, WEO-PMB and BPMB mixtures.

Sample	Stiffness Modulus [MPa] at 23 °C	Stiffness Modulus [MPa] at 10 °C	Stiffness Modulus [MPa] at −2 °C
PMB	6249	11,518	19,743
WEO-PMB	5213	9777	18,273
BPMB	5191	10,346	19,743

## Data Availability

Not applicable.
